# Apoptosis-targeted gene therapy for non-small cell lung cancer using chitosan-poly-lactic-co-glycolic acid -based nano-delivery system and CASP8 and miRs 29A-B1 and 34A

**DOI:** 10.3389/fbioe.2023.1188652

**Published:** 2023-06-06

**Authors:** Sourav Chattopadhyay, Shashanka Shekhar Sarkar, Sheetanshu Saproo, Sheetal Yadav, Deepika Antil, Bodhisatwa Das, Srivatsava Naidu

**Affiliations:** Department of Biomedical Engineering, Indian Institute of Technology Ropar, Rupnagar, Punjab, India

**Keywords:** nano-formulations, MicroRNAs, gene delivery, non-small cell lung cancer, combinatorial gene therapy, tumor spheroids

## Abstract

Non-small cell lung cancer (NSCLC) is a leading cause of cancer-related deaths worldwide, with resistance to apoptosis being a major driver of therapeutic resistance and aggressive phenotype. This study aimed to develop a novel gene therapy approach for NSCLC by targeting resistance to apoptosis. Loss of function mutations of caspase 8 (CASP8) and downregulation of microRNAs (miRs) 29A-B1 and 34A were identified as key contributors to resistance to apoptosis in NSCLC. A biodegradable polymeric nano-gene delivery system composed of chitosan-poly-lactic-co-glycolic acid was formulated to deliver initiator CASP8 and miRs 29A-B1 and 34A. The nano-formulation efficiently encapsulated the therapeutic genes effectively internalized into NSCLC cells and induced significant apoptosis. Evaluation of the nano-formulation in A549 tumor spheroids showed a significant increase in apoptosis within the core of the spheroids, suggesting effective penetration into the spheroid structures. We provide a novel nano-formulation that demonstrate therapeutic potential for suicidal gene therapy in NSCLC.

## 1 Introduction

Non-small cell lung cancer (NSCLC) is the most prevalent histological subtype of lung cancer, accounting for about 85% of cases (Thandra et al., 2021) with a dismal 5-year survival rate of 10%–20% (Min and Lee, 2021). The incidence and mortality rates of NSCLC are alarmingly high, exceeding those of colon, prostate, and breast cancer combined (Chen et al., 2020). Despite the initial effectiveness of cytotoxic chemotherapy regimens involving platinum compounds and taxanes (Baxevanos and Mountzios, 2018), the therapeutic management of NSCLC faces significant challenges due to severe side effects, drug resistance, and disease relapse (Ashrafi et al., 2022). Targeted therapy and immunotherapy have emerged as promising approaches by selectively targeting oncogenic alterations in NSCLC that impair tumor cell proliferation and other cancer hallmarks (Konig et al., 2021). However, resistance to these treatments due to survival mutations and tumor heterogeneity limits their efficacy (Lim and Ma, 2019). Thus, innovative and more effective treatment approaches are urgently needed to overcome these obstacles.

Extensive molecular profiling of NSCLC tumors has unveiled a complex and heterogeneous landscape of genetic aberrations that contribute to the development and progression of the disease (Bradner et al., 2017). Emerging evidence has demonstrated that the loss of apoptosis is a crucial factor in the development of therapeutic resistance (Pommier et al., 2004), aggressive phenotypes, and poor prognosis (Singhal et al., 2005) in NSCLC patients. Dysregulation of several genes involved in the regulation of apoptosis, such as IAP, NR4A1 (Lee et al., 2020), and SPRR3 (Li et al., 2020), have been identified in NSCLC. Notably, suppression of caspases, which are key enzymes involved in the initiation and execution of apoptosis, has been attributed to chemosensitivity in NSCLC (Zuo et al., 2020). Furthermore, genetic polymorphisms have been identified as a critical factor in conferring resistance to apoptosis in NSCLC (Szczyrek et al., 2021). Nonetheless, these findings underscore potential for targeted therapies aimed at restoring apoptosis as a promising strategy for treating this deadly disease.

MicroRNAs (miRs) are small, single-stranded RNA molecules that negatively regulate target mRNAs by sequence-specific degradation or translational inhibition (Chong et al., 2021). Dysregulated miR expression has shown to play crucial role in tumorigenesis, cancer progression, and resistance to various therapeutic regimens (Chandra et al., 2017; Yan et al., 2022). The miR29 cluster is a group of tumor suppressor miRs that play a vital role in regulating cell proliferation, cell cycle progression, and other cancer-related processes (Kwon et al., 2019). Downregulation of miR29A-B1 is commonly observed in various cancer types and is associated with poor patient outcomes (Kwon et al., 2019). MiR29A-B1 targets several anti-apoptotic genes, including MCL1, which promotes cancer cell survival and proliferation (Mott et al., 2007), and CDC42, a member of the Rho protein family that plays a crucial role in cytoskeletal organization and cell cycle regulation (Zhang et al., 2016). Additionally, miR29A-B1 negatively regulates LIM and SH3 protein 1 and CDC42 in NSCLC, indicating its tumor suppressor function (Hu et al., 2016). CDK6, another target of miR29A-B1, causes G1-phase arrest in the cell cycle, further highlighting its importance in regulating cancer-related processes (Zhao et al., 2010). Further, miR34A was the first miRNA to be discovered as a target of the tumor suppressor p53 and plays a crucial role in apoptosis regulation, by inhibiting the expression of genes that promote cell proliferation, miR34A synergizes with p53 to promote apoptosis (Shirjang et al., 2019; Xiong et al., 2019). In NSCLC, miR34A has been shown to target several proteins such as BCL2, SIRT1, Survivin, and MDM4, ultimately promoting apoptosis (Farooqi et al., 2017). Additionally, low levels of miR34A have been associated with poor overall survival in NSCLC patients (Zhao et al., 2017). Similarly, dysregulated expression of miR29A-B1 and miR34A have been implicated in NSCLC (Liu et al., 2018; Kim et al., 2019). Therefore, supplementing miR29A-B1 and miR34A via gene therapy could be a promising strategy to restore apoptosis in NSCLC.

Gene therapy (GT) involves the manipulation of genetic material within pathological cells or tissues to correct or restore gene function for therapeutic purposes (Kaufmann et al., 2013). Compared to conventional cancer therapies, GT offers several advantages, including simultaneous targeting of multiple oncogenic pathways, reduced toxicity, and longer-lasting effects (Das et al., 2015). Despite its potential, GT still faces various technical and scientific hurdles that are hindering its clinical application. These hurdles include the development of safe and effective gene delivery vectors, targeted delivery to specific cells and tissues, minimizing off-target effects, and understanding the long-term consequences of GT (Pan et al., 2021). The delivery of therapeutic nucleic acids typically involves either viral or non-viral vectors (Nayerossadat et al., 2012). While viral vectors are commonly used due to their efficacy, they are limited by drawbacks such as host immune response, integration into the host genome, limited cargo capacity, and biosafety concerns (Robbins and Ghivizzani, 1998). Non-viral vectors, such as liposomes or nanoparticles (NPs), offer an alternative approach for delivering therapeutic genes (Ramamoorth and Narvekar, 2015). These vectors have advantages such as low immunogenicity, low toxicity, and ease of manufacturing. Recent advances in nanotechnology have allowed for the customization of NPs for gene delivery, resulting in improved cargo capacity, specificity, encapsulation for stability, and enhanced safety of GT (Miron-Barroso et al., 2021). Notably, biodegradable nanoparticles particularly those composed of poly-lactic-co-glycolic acid (PLGA) have been widely studied in gene delivery due to their biocompatibility and ability to be naturally reabsorbed (Ramezani et al., 2017; Rezvantalab et al., 2018). Furthermore, Chitosan has also been widely investigated as a potential gene delivery vector, as it possesses a cationic charge that can effectively bind and condense DNA, and other properties such as mucoadhesion, biodegradability, and the ability to aid the penetration of large molecules through mucosal surfaces (Illum, 1998; Panyam et al., 2002).

In this study, a gene delivery method utilizing chitosan-PLGA was developed to deliver the tumor suppressor miR29A-B1 and miR34A, along with CASP8, for NSCLC therapy. The results demonstrated that the chitosan-PLGA-based gene delivery modality was effective in delivering the therapeutic genes into NSCLC cell line and tumor spheroids, resulting in a significant increase in apoptosis. The use of tumor suppressor miR29A-B1 and miR34A, as well as CASP8, highlights the importance of targeting multiple pathways in NSCLC therapy.

## 2 Materials and methods

### 2.1 Computational analysis

The Cancer Genome Atlas (TCGA) read count data for lung adenocarcinoma (LUAD) was retrieved from cBioPortal (https://www.cbioportal.org/). The Gene Set Enrichment Analysis (GSEA) was performed on the retrieved LUAD data using GSEA online analysis tool (https://nasqar.abudhabi.nyu.edu/ClusterProfShinyGSEA/). MiR expression profiles for LUAD patients and adjacent normal tissue were obtained from GEO (Gene Expression Omnibus) dataset GSE169587 and the differential expression was analyzed using GEO2R web tool (https://www.ncbi.nlm.nih.gov/geo/geo2r/). The statistical significance was determined using Benjamini and Hochberg False discovery rate ≤0.05. The mutation profile of CASP8 and survival analysis of LUAD patients with CASP8 and miR29A-B1 and miR34A alterations were analyzed using cBioportal.

### 2.2 Synthesis of chitosan-PLGA nanoparticles

Plasmids expressing miR29A-B1 (Cat no: 64231, size 8.8 kb), miR34A (Cat no: 78125, size 5.53 kb), and CASP8 (Cat no: 11817, size 6.85 kb) were purchased from Addgene. The emulsion-evaporation technique was used for fabrication of chitosan-PLGA NPs. Briefly, 10 mL of ethyl acetate was mixed to 200 mg of PLGA at room temperature (RT) until it dissolved completely. The solution was further mixed with an aqueous stabilizer mixture containing 100 mg polyvinyl alcohol (PVA), 30 mg chitosan dissolved in acidic solution, and CASP8 alone (50 µg/mL) or in combination with miR34A (25 µg/mL) and miR29A-B1 (25 µg/mL), (in a ratio of 2:1:1 respectively), in 10 mL water at RT with continuous stirring for 3 h. Next, emulsification was performed by sonication for 20 min. The residual ethyl acetate was removed from the mixture by heating at 40°C on a magnetic stirrer.

### 2.3 Characterization of chitosan-PLGA nanoparticles

The size distribution of the chitosan-PLGA NPs, including hydrodynamic size and average particle size distributions were determined using dynamic light scattering (DLS) (Microtrac/nanotrack flex) at 25°C. The diameter of dried state NPs and surface topography were evaluated by atomic force microscopy (AFM) (Multimode 8, Bruker, United States). The size and shape of NPs were determined using scanning electron microscope (JEOL 6610LV, JEOL, Japan). For Fourier-transform infrared (FTIR) spectroscopy, PLGA, PVA, and chitosan-PLGA NPs were frozen at −80°C for 2 h before lyophilization. The spectrum was recorded in 4,000–400 cm^−1^ range using FTIR spectrophotometer (Bruker Tensor 27, United Kingdom).

### 2.4 Quantitative determination of the encapsulated plasmid DNA

1 mL (about 100 µg/mL in total) of CASP8+miR34A + miR29A-B1 encapsulated chitosan-PLGA NPs were dissolved in Dulbecco’s phosphate-buffered saline (DPBS) and centrifuged at 10,000 rpm for 10 min to separate the particles. The retained particle pellets were dissolved in 50 µL of chloroform for 10 min at RT. Next, 500 µL of isopropyl alcohol was added to the extract and centrifuged at 13,000 rpm for 30 min. The amount of DNA extracted from NPs was measured using a NanoDrop spectrophotometer (Thermo Fisher Scientific, Waltham, Massachusetts, United States).

### 2.5 Cell culture

Human LUAD cell line, A549 was purchased from National Center for Cell Sciences, Pune, India. The cells were cultured in RPMI 1640 medium (Gibco, Thermo Fisher Scientific) supplemented with 10% fetal bovine serum (Gibco), and 1% penicillin-streptomycin (Gibco) at 37°C with 5% CO_2_.

### 2.6 Biocompatibility of chitosan-PLGA nanoparticles

A549 cells were seeded in a six-well plate (1 × 10^5^ cells/well) and treated with 10, 20, 40, and 80 µg/mL chitosan-PLGA NPs (in serum-free media), respectively or an equivalent volume of serum-free media only as control. After 48 h of incubation, cells were trypsinized and resuspended in sheath fluid. Next, the treated cells were stained with PI and acquired using flow cytometer (BD Accuri C6 plus). The data was analyzed using FlowJo software (BD Biosciences).

### 2.7 Chitosan-PLGA nanoparticles DNA release assay

Chitosan-PLGA NPs were resuspended in Tris buffer and pH gradient were created with either 6N HCl or 6N NaOH.

### 2.8 Visualization of cellular uptake of chitosan-PLGA nanoparticles

1 × 10^5^ A549 cells were seeded per well of a 6 well plate, and incubated with chitosan-PLGA NPs encapsulated with red fluorescence protein expression plasmid (RFP) (100 µg/mL) at a final concentration of 80 µg/mL (in serum-free media) for 5 h at 37°C. The cells were gently washed with DPBS, replenished with complete media, and incubated for another 48 h. For a comparison, A549 cells were transiently transfected with RFP (100 µg/mL) for 48 h using Lipofectamine 2000 reagent according to the manufacturer’s instructions (Thermo Fisher Scientific), as a control A549 cells were incubated with RFP (100 µg/mL) without transfection reagent or NPs. After 48 h, cells were washed with DPBS, fixed, and imaged using fluorescence microscope (Leica DMi8, Leica Microsystems, Wetzlar, Germany).

### 2.9 Apoptosis activity of the plasmid DNA coated chitosan-PLGA nanoparticles

A549 cells were treated with 80 µg/mL (in serum-free media) of chitosan-PLGA NPs alone or in combinations with CASP8 and miRs, along with empty vector control, for 48 h. The cells were trypsinized, resuspended in sheath fluid, and stained with Annexin V and propidium iodide (PI) as per the manufacturer’s instructions (BD Annexin-V/PI apoptosis detection kit, BD Biosciences, New Jersey, United States) followed by flow cytometry (BD Accuri C6 plus, BD Biosciences).

### 2.10 Ethidium bromide and acridine orange staining

Apoptosis/necrosis potential of the chitosan-PLGA NPs was assessed using ethidium bromide (EtBr)/acridine orange (AO) staining. In brief, following 48 h incubation of A549 cells with different concentrations of chitosan-PLGA NPs, the cells were washed and stained with EtBr and AO (200 μg/mL; in PBS). Live cells with intact cell membranes were stained with green (AO). In contrast, cells with membrane pores or ruptured cell membranes allowed EtBr diffusion into the cell cytosol and characterized by red fluorescence. Images were captured at a magnification of ×10 and the quantitative mean fluorescence values were retrieved using fluorescence microscope (Leica DMi8, Leica Microsystems). The cell number was quantified using ImageJ software.

### 2.11 Validation of apoptotic potential of the formulation in A549 tumor spheroids

A549 spheroids were prepared according to previously published protocol with modifications (Friedrich et al., 2009). Briefly, 1 × 10^3^ A549 cells were seeded into the agarose-coated 96 well plates. The medium was changed every alternate day. Spheroids were maintained for 10 days and selected based on size. The spheroids were then treated with chitosan-PLGA NPs or CASP8 alone or miRs alone or in combinations with CASP8 and miRs for 48 h. Untreated control spheroids were treated with serum-free media without NPs. After 48 h of incubation, spheroids were stained with Annexin-V/PI stain as per the kit instructions (BD Annexin-V/PI apoptosis detection kit), and images were captured and the mean fluorescence intensities were quantified using built-in software of Leica DMi8 fluorescence microscope.

### 2.12 RNA isolation and quantitative PCR (qPCR)

A549 cells and spheroids were treated with 80 µg/mL chitosan-PLGA NPs for 48 h, and total RNA was isolated using TRIzol reagent (Ambion, Thermo Fisher Scientific, Waltham, MA, United States). For miR expression analysis, 100 ng of total RNA was reverse transcribed to cDNA using universal primers and one-step qPCR. For gene expression analysis, 1,000 ng of total RNA was reverse transcribed using PrimeScript^TM^ 1st strand cDNA Synthesis Kit (TaKaRa Biotechnology Co Ltd.), and qPCR was performed using TB Green^®^ Premix Ex Taq™ (Tli RNaseH Plus) (TaKaRa Biotechnology, Japan). Gamma-actin or U6 were used as normalizers. The relative RNA levels were determined using the 2^−ΔΔCT^ method. Details of primers used are provided in Supplementary Table S1.

### 2.13 Statistical analysis

All experiments were carried out as three independent replicates. Results represent as the mean ± standard error mean (SEM). The Student’s *t*-test measured significant differences between the two groups. A *p*-value of ≤0.05 was selected as significance. Bar graphs were prepared using GraphPad Prism (GraphPad Software Inc, CA, United States).

## 3 Results and discussion

### 3.1 Genetic alterations in apoptotic CASP8 and miRs in NSCLC

**FIGURE 1:**
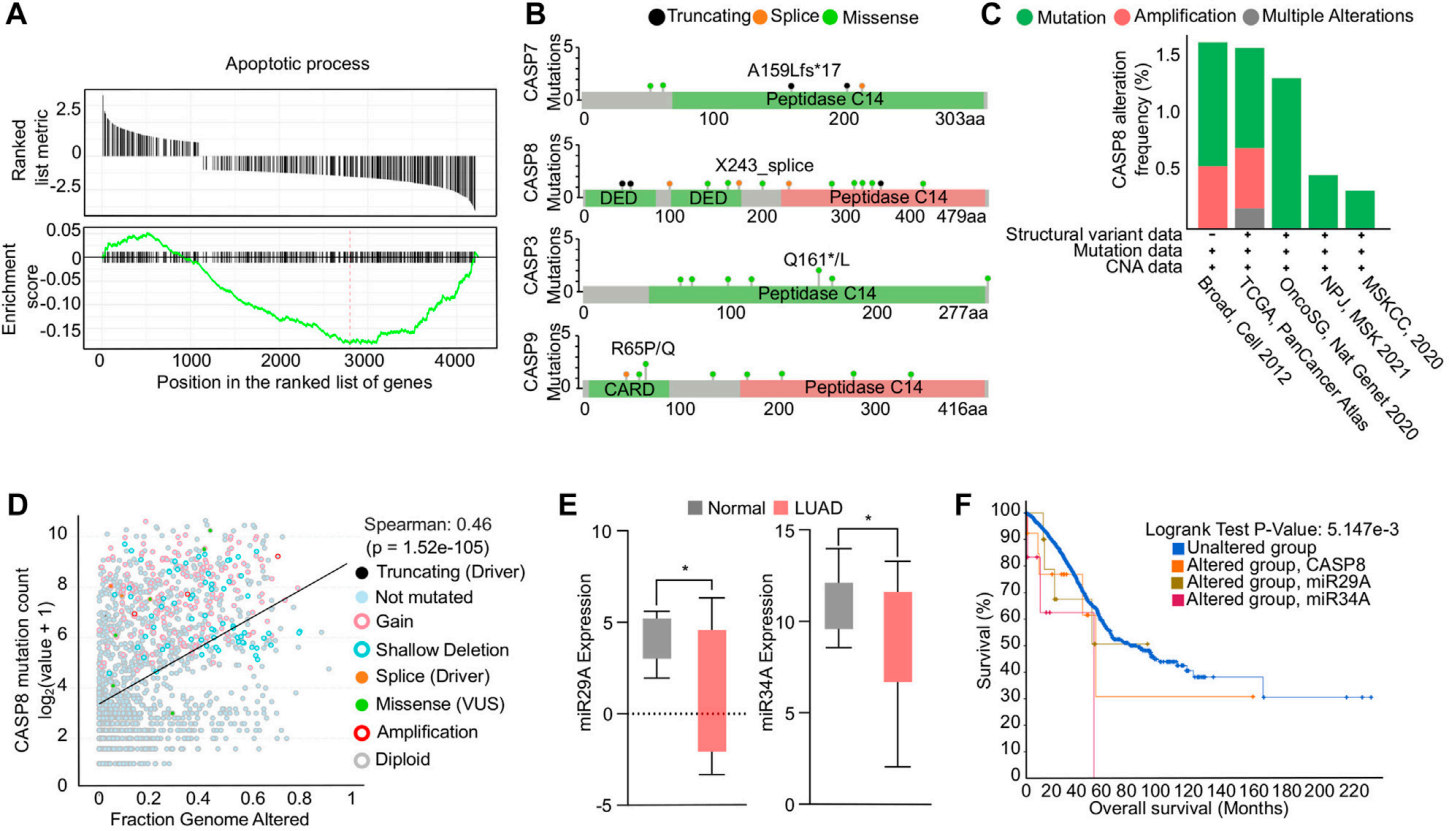
Functional analysis of apoptosis in NSCLC. **(A)** GSEA enrichment plot showing negative association of genes involved in apoptosis in NSCLC. **(B)** Mutational profiles of caspase genes in NSCLC. **(C)** Bar graph showing the genetic alterations in CASP8 gene in multiple NSCLC transcriptomic datasets. **(D)** Correlation analysis showing a positive correlation between CASP8 mutation and fraction genome altered. **(E)** Box plots showing downregulation of miR29A-B1 and miR34A expression in NSCLC compared to adjacent normal tissue, *p < 0.05. **(F)** Survival plot showing reduced overall survival in patients with alterations in miR29A-B1, miR34A, and CASP8.

Impaired apoptosis in NSCLC can be attributed to dysregulation of genes involved in the apoptotic pathways (Lee et al., 2020). To determine the global genetic alterations and its impact on apoptosis in NSCLC, we conducted a GSEA analysis using TCGA NSCLC transcriptome data. The analysis revealed a significant downregulation of the apoptosis hallmark, indicated by a negative enrichment score (Figure 1A). We further investigated the mutation profiles of caspases in NSCLC patients using data available on cBioPortal. Our analysis revealed that CASP8, the effector molecule of apoptosis, had a higher mutation load compared to other caspases. Specifically, our analysis of mutation profiles identified multiple loss-of-function mutations in the death effector domain and peptidase domain of the CASP8 protein, which may enable cells to evade apoptosis (Figure 1B). In addition, our investigation into the genetic alterations associated with NSCLC has revealed a high frequency of alterations in the CASP8 gene, including mutations and structural variations, across various TCGA-NSCLC datasets (Figure 1C). Moreover, through Spearman correlation analysis, we have observed a positive correlation between the mutation profiles of the CASP8 gene and genomic alterations, suggesting that these mutations play a significant role in evading apoptosis, thus contributing to the accumulation of oncogenic mutations in NSCLC (Figure 1D). Notably, genetic variations and polymorphisms in the CASP8 gene have been implicated in the development of NSCLC (Szczyrek et al., 2021). In addition, decreased CASP8 expression has been strongly correlated with chemoresistance in NSCLC (Okouoyo et al., 2004; Paul et al., 2012). These findings underscore the importance of CASP8 as a potential therapeutic target in the treatment of NSCLC. Next, we specifically analyzed the expression profiles of pro-apoptotic miRs miR29A-B1, and miR34A in TCGA NSCLC transcriptome data. Our analysis showed a significant downregulation of pro-apoptotic miRs in NSCLC patients compared to adjacent normal tissues (Figure 1E). Furthermore, Kaplan-Meier survival analysis demonstrated a significant reduction in overall survival of NSCLC patients with alterations in miR29A-B1, miR34A, and CASP8 (Figure 1F). Epigenetic modifications, including DNA methylation, play a crucial role in cancer development and progression, and promoter methylation-induced silencing of tumor suppressor genes is a well-known hallmark feature of oncogenesis (Ilango et al., 2020). Li et al. (2012), Li et al. (2012) reported the downregulation of the miR29 family association with the epigenetic silencing of the pro-apoptotic gene PTEN expression. In addition, studies have shown that the reduced levels of miR29A-B1 in cancer may result in the upregulation of DNA methyltransferases, DNMT3A and DNMT3B (Morita et al., 2013), which subsequently lead to enhanced DNA methylation and downregulation of tumor suppressor gene expression. Notably, miR29A family has shown to have several genes including BCL2 that have anti-apoptotic functions (Bargaje et al., 2012). In addition, miR29A-B1 reconstitution has been shown to inhibit cell proliferation and induced cell cycle arrest in gastric cancer via p42.3 targeting (Cui et al., 2011), and reduces migration and invasion of pancreatic cancer cells via suppressing LOXL2 expression (Dey et al., 2020). Conversely, loss of miR29A-B1 expression has been linked to melanoma tumor development and poor prognosis (Vera et al., 2021). The dysregulation of miR34A has also been implicated in the inhibition of apoptosis in NSCLC. miR34A is a direct target of the tumor suppressor protein p53 and its downregulation has been shown to upregulate anti-apoptotic genes such as BCL2, cyclin D1, cyclin E2, and SIRT-1, thereby inhibiting apoptosis. In addition, restoration of miR34A has shown to inhibit metastasis in pancreatic cancer (Nalls et al., 2011) and gastric cancer (Peng et al., 2014). Furthermore, ectopic expression of miR34A reduced cancer stem cell properties, enhanced the chemosensitivity and reduced tumor burden in breast cancer (Park et al., 2014). These findings suggest that the concomitant downregulation of CASP8, miR29A-B1, and miR34A results in an overall decrease in the apoptosis process in NSCLC (Hermeking, 2010). Reconstituting the expression of these genes, could impact multiple regulatory networks involved in the apoptosis process and subsequently restore apoptosis in NSCLC.

### 3.2 Synthesis and physicochemical characterization of chitosan-PLGA nanoparticles

**FIGURE 2:**
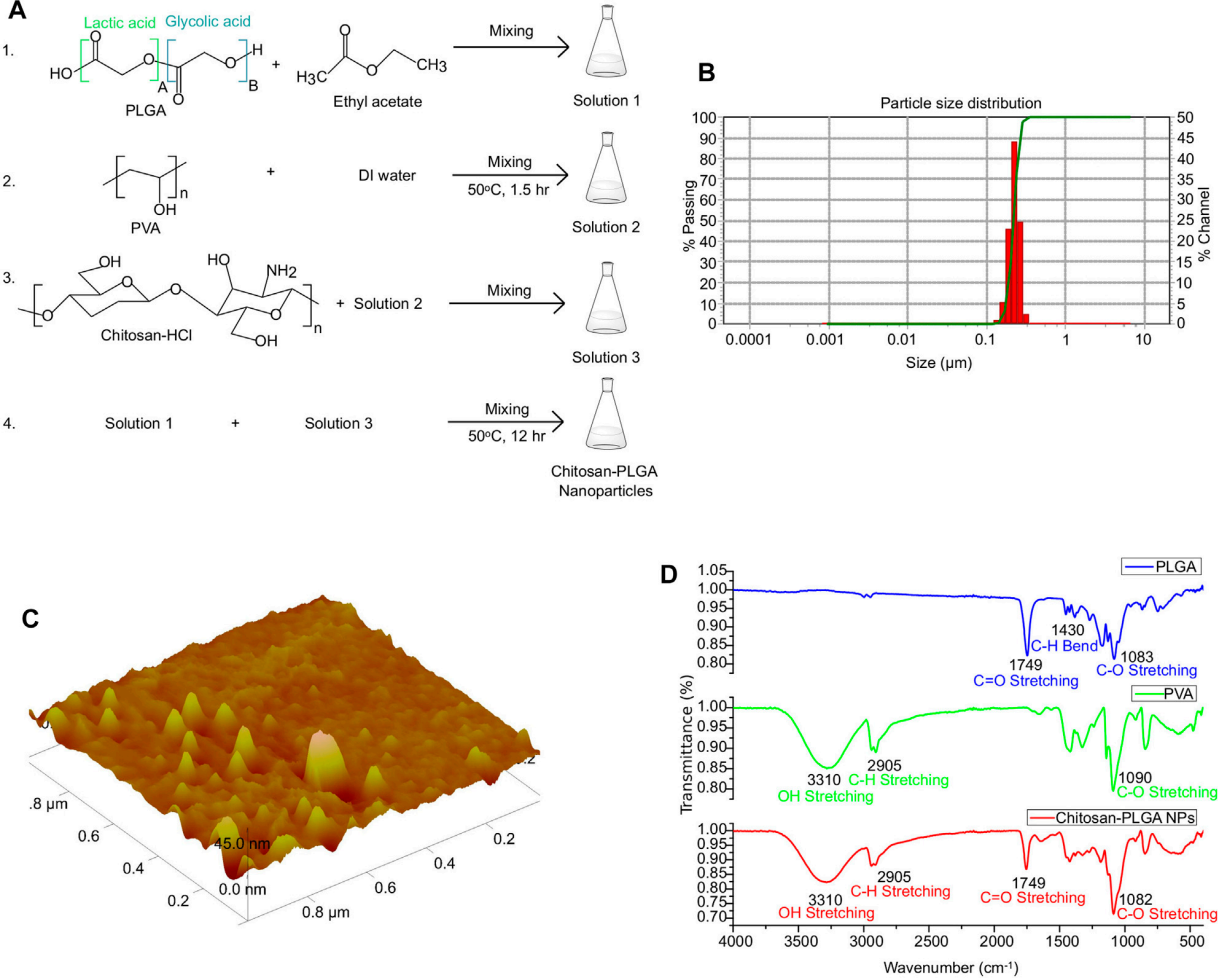
Synthesis and characterization of chitosan-PLGA-NPs. **(A)** Scheme showing chitosan-PLGA-NPs preparation. **(B)** Particle distribution plot (representative of 3 independent experiments) showing the hydrodynamic particle size distribution. **(C)** Atomic Force Microscopy image showing dry-state particle size of chitosan-PLGA-NPs. **(D)** Fourier transform infrared spectroscopy peaks showing functional groups in the synthesized chitosan-PLGA NPs corresponding to PLGA and PVA.

PLGA, an FDA-approved biomaterial for drug delivery, is composed of lactic acid and glycolic acid. PLGA-NPs, which are biocompatible and biodegradable, are ideal for therapeutic gene delivery due to their reduced toxicity (Cappellano et al., 2019; Wang et al., 2021). In this study, we synthesized chitosan-PLGA NPs using the emulsion diffusion and evaporation technique with a mixture of chitosan and PVA as a stabilizer. This stabilizer acted as a barrier between the PLGAs, preventing self-aggregation of the NPs and resulting in an oil in water emulsion form. The PVA chitosan blend possess a net positive surface charge due to the amine groups in the chitosan molecule. At acidic pH, these amine groups become protonated, which further increases the positive charge density of the blend (Mikusova and Mikus, 2021). Additionally, at acidic pH, the negatively charged carboxylic groups of PLGA can electrostatically interact with the negatively charged head groups on the cell membrane, allowing for the attachment of the NPs to the membrane, and internalization into the cells (El-Hammadi and Arias, 2022). The scheme for the synthesis of chitosan-PLGA NPs is shown in Figure 2A. Accurate characterization of the physicochemical properties of NPs is crucial for determining their release properties, stability profile, and efficacy in cellular uptake (Mahmoudi, 2021). The size of NPs, in both their hydrodynamic and dry state, plays a critical role in mediating their function. To evaluate the size and homogeneity of the PLGA NPs, we used AFM for 3D characterization and DLS for hydrodynamic diameter evaluation. DLS analysis revealed a hydrodynamic diameter of 139 nm with a polydispersity index (PDI) of 0.2, suggesting a homogeneous distribution in size (Figure 2B). AFM imaging showed the dry size of the NPs to be approximately 45 nm (Figure 2C). The primary mechanism for producing NPs is believed to be the formation of irregularly sized globules of the solvent dispersed in equilibrium with the continuous phase during stirring. These globules provide a large interface for the stabilizer (PVA) to be absorbed onto. Smaller globules are also produced during homogenization. The addition of water and the heating step disrupt the equilibrium, causing the organic solvent to diffuse to the outer surface. As a result, new smaller globules, which are less than 50 nm in size in the dry state as seen in AFM, are created as the solute is transported. The heating step also helps to create a final colloidal suspension that is more uniform in size and free of ethyl acetate. Further, FTIR spectroscopy analysis confirmed the composition of the PLGA, PVA, and PLGA NPs, and the peaks corresponding to different functional groups were shown in Figure 2D. In addition, SEM images confirm the size distribution of the NPs (Supplementary Figure S1). The distinct measurement principles of AFM, DLS, and SEM could contribute to the differences in size measurements obtained. AFM can provide high-resolution images of individual particles, but its results may be influenced by particle orientation and surface adsorption. DLS measures hydrodynamic size, which is affected by particle shape and surface charge. SEM provides morphological information, but the sample preparation may introduce artifacts or modify the surface features of the particles (Eaton et al., 2017). In conclusion, the combination of AFM, DLS, and SEM measurements can provide a more comprehensive characterization of particle properties, including size, shape, surface features, and aggregation state.

### 3.3 Chitosan-PLGA NPs release property, cellular internalization, and biocompatibility analysis

**FIGURE 3:**
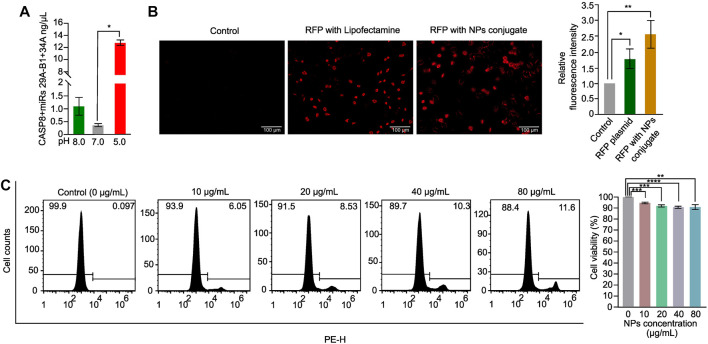
Chitosan-PLGA-NPs DNA release property and cytotoxicity evaluation. **(A)** Bar graph showing the DNA release property of the chitosan-PLGA-NPs at different pH points evaluated using spectrophotometer (*p ≤ 0.05). **(B)** Fluorescence microscopy images showing internalization efficiency of the chitosan-PLGA-NPs coated with RFP plasmid, compared to lipofectamine (Control) and RFP plasmid alone, right bar graph showing relative fluorescence intensity. **(C)** Histograms showing toxicity profiles of the chitosan-PLGA-NPs at different concentrations, right bar graph showing percentage cell viability. All the experiments were performed in biological triplicates.

The success of NPs in drug delivery is dependent on their release property and biocompatibility (Mahmoudi, 2021). To evaluate the release property of the CASP8 expression vectors and miRs miR29A-B1 and miR34A encapsulated in chitosan-PLGA NPs, we employed spectrophotometric measurement of DNA release at different pH levels. The findings revealed that there was a significant release of DNA from the NPs at pH-5.0 compared to other pH conditions (Figure 3A). Previous studies have shown that PLGA-NPs are efficient endosome-mediated intracellular delivery vehicles (Cartiera et al., 2009). Under acidic conditions of the endosome, the protonation of the amine groups on the surface of the NPs increases. This protonation disrupts the electrostatic interactions between the positively charged amine groups on the NP surface and the negatively charged phosphate groups in the DNA backbone. As a result, the DNA is released from the NPs, which leads to a higher yield of released DNA at lower pH values (Galliani et al., 2019). Thus, the maximum endosome release of NPs carrying DNA cargo can be achieved with chitosan-PLGA NPs in an acidic pH environment, as evidenced by the increase in DNA release at pH-5.0. We conducted transient transfection experiments using an expression vector containing the RFP reporter gene as a readout to determine the internalization capacity of chitosan-PLGA NPs without any transfection reagent. We compared the results with A549 cells transfected with the RFP plasmid using lipofectamine and a control group consisting of pure DNA without transfection reagent and NPs. Fluorescence microscopy results showed efficient uptake of the chitosan-PLGA NPs in A549 cells with a significant RFP expression after 48 h of incubation, as compared to the lipofectamine-transfected RFP plasmid and the control group, and did not find any comparable morphological changes in cells between lipofectamine and NPs conditions (Figure 3B). The RFP expression in cells treated with the NPs indicate that the DNA cargo was not damaged during the heating of the NP emulsion at 40°C. Since the typical melting temperature of nucleic acids of this size is considerably higher (Khandelwal and Bhyravabhotla, 2010) than the temperature used in the experiment, the results suggest that the DNA cargo retained its functional integrity. Furthermore, the biocompatibility of the chitosan-PLGA NPs was evaluated by measuring cytotoxicity at various NPs concentrations in A549 cells using PI-staining. The flowcytometry analysis indicated that a maximum dose of 80 µg/mL yielded more than 88% viable cells, indicating the high biocompatibility of chitosan-PLGA NPs (Figure 3C). Overall, the results suggest that the NPs have low toxicity at higher concentrations and release DNA at acidic pH-5.0. The low toxicity of the chitosan-PLGA NPs can be attributed to their hydrolysis into lactic acid and glycolic acid, which are biocompatible monomers metabolized via the tricarboxylic acid cycle (Makadia and Siegel, 2011). In addition, chitosan is a biocompatible and biodegradable polymer (Dodane et al., 1999), that can bind to negatively charged DNA through its amine groups, and protects DNA from nucleases (Artursson et al., 1994; Schipper et al., 1996). Chitosan can also attach to cell membranes and enhance paracellular permeability, making it a promising nanocarrier. Our chitosan-PLGA NPs successfully delivered our gene of interest inside cells and showed significant expression of RFP, indicating its potential to overcome intracellular trafficking barriers.

### 3.4 Chitosan-PLGA NPs encapsulating CASP8 and miRs induced apoptosis in A549 cells and tumor spheroids

**FIGURE 4:**
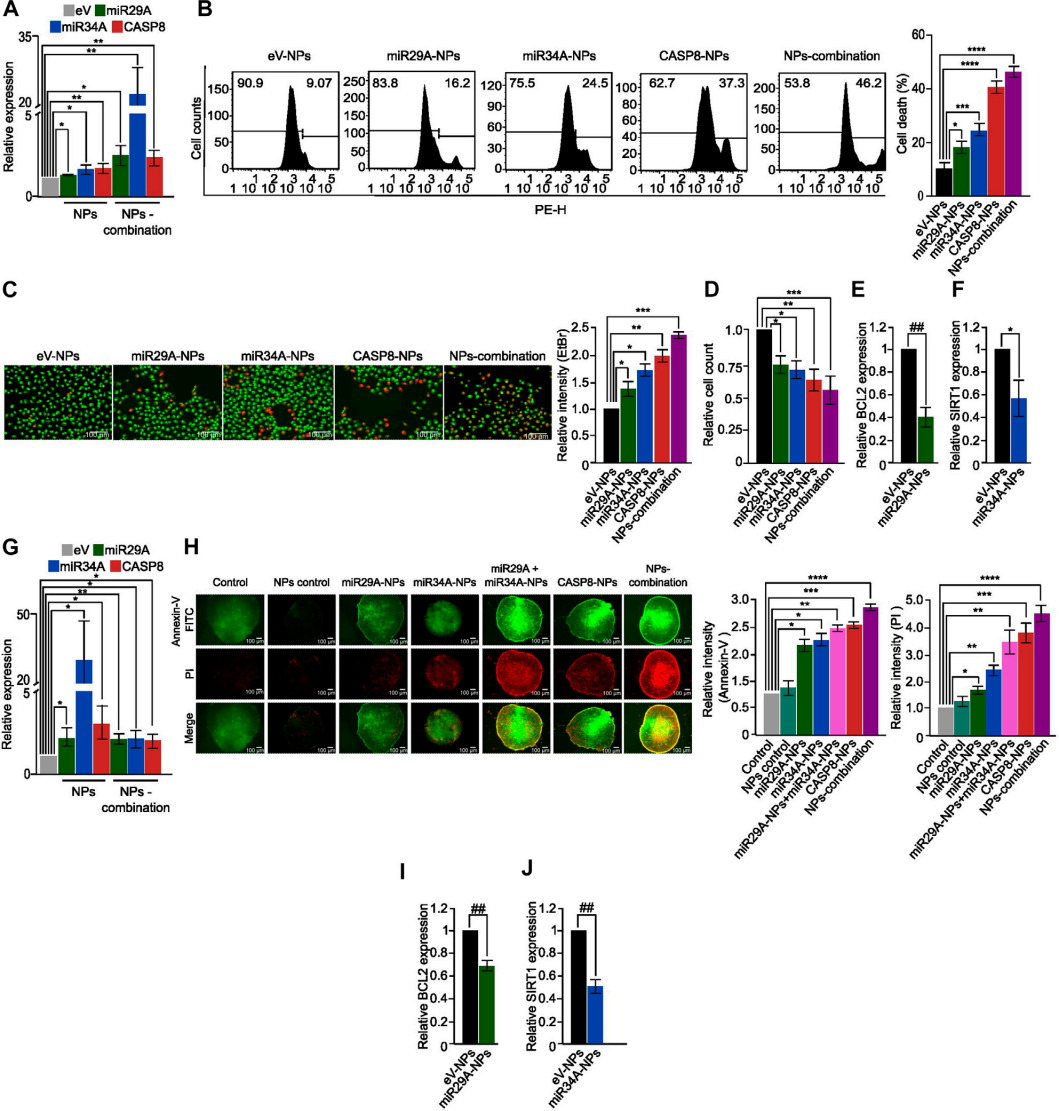
Apoptotic potential of the nano-formulation in A549 cells and spheroids. **(A)** Bar graphs showing relative expression of miR29A, miR34A and CASP8 in A549 cells treated with miR29A-NPs/miR34A-NPs/CASP8-NPs, respectively or a combination of miR29A, miR34A and CASP8 NPs (NPs-combination), compared to control. **(B)** Histograms showing increased apoptosis in A549 cells treated with miR29A-NPs/miR34A-NPs/CASP8-NPs or NPs-combination compared to controls; bar graph showing percentage of cell death. **(C)** Fluorescence microscopy images showing increased apoptosis in A549 cells treated with miR29A-NPs/miR34A-NPs/CASP8-NPs or NPs-combination, compared to controls, bar graph showing relative intensity of ethidium bromide (EtBr), **(D)** Bar graph showing relative cell count for panel. Bar graphs showing relative expression of miR29A target BCL2 **(E)** and miR34A target SIRT1 **(F)** in A549 cells treated with miR29A-NPs or miR34A-NPs respectively, compared to controls. **(G)** Bar graphs showing relative expression of miR29A, miR34A and CASP8 in A549 spheroids treated with miR29A-NPs/miR34A-NPs/CASP8-NPs or NPs-combination, respectively. **(H)** Fluorescence microscopy images showing increased Annexin-V FITC and propidium iodide (PI) staining in A549 spheroids treated with miR29A-NPs/miR34A-NPs/CASP8-NPs or NPs-combination, compared to controls, bar graph showing relative intensity of Annexin-V and PI. Bar graphs showing relative expression of BCL2 **(I)** and SIRT1 **(J)** in A549 spheroids treated with miR29A-NPs or miR34A-NPs respectively, compared to controls. All the experiments were performed in biological triplicates. eV = empty vector. *p ≤ 0.05, **p ≤ 0.005, ***p ≤ 0.0005, ****p ≤ 0.00005, ##*p* ≤ e−6, ###*p* ≤ e−8, $*p* ≤ e−10.

To evaluate the effectiveness of chitosan-PLGA NPs as a GT delivery system, we first treated A549 cells with chitosan-PLGA-NPs encapsulated with miR29A-B1, miR34A and CASP8 plasmids either separately or in combination, and measured their expression. QPCR analysis showed a significant upregulation of these genes either separately or in combination when compared to control (Figure 4A). Next, the effect of the formulation on apoptosis was assessed by PI staining—flow cytometry analysis. The findings indicated that concomitant supplementation of miR29A-B1 and miR34A with CASP8-NPs formulation resulted in 51% cell death, while the use of miR29A-B1, miR34A, or CASP8 alone carrying NPs resulted in 19.5%, 23%, and 38.2% apoptosis, respectively (Figure 4B). To further validate the apoptotic induction capability of chitosan-PLGA NPs, we performed EtBr/AO staining and microscopy. The results showed that the group treated with CASP8-NPs along with miR29A-B1/miR34A exhibited a significant increase in cell death compared to the groups treated with individual components (Figures 4C,D). It is known that the anti-apoptotic proteins BCL2 and SIRT1 are targeted by miR29A-B1 and miR34A, respectively (Bargaje et al., 2012). In light of this, we assessed the impact of miRs encapsulated in chitosan-PLGA NPs on the expression of BCL2 and SIRT1 in A549 cells. Our qPCR analysis indicated a significant decrease in the expression of these genes upon treatment with miR29A-B1 (Figure 4E) or miR34A NPs (Figure 4F). These findings provide evidence that chitosan-PLGA NPs are effective in delivering gene constructs to induce apoptosis in A549 cells, and that the combination of miRs with the CASP8 gene has a synergistic apoptotic induction potential. The ability of nanocarrier-mediated drug delivery to penetrate tumors is a critical factor for its efficacy (Zein et al., 2020; Bie et al., 2022). Tumor spheroids can mimic the physiological properties of tumors, including a rapid proliferation rate at the periphery, a necrotic core at the center, and the presence of a nutrient gradient across the spheroid (Pinto et al., 2020). Therefore, we tested the uptake and penetration capacity of our nano-formulation using an A549 spheroid model. Chitosan-PLGA NPs loaded with miR29A-B1, miR34A, CASP8, or combinations of these genes were administered to A549 spheroids, and the expression of these genes was assessed. Notably, similar to the A549 cell line, a significant increase in gene expression was observed in response to the treatment with these NPs (Figure 4G). In addition, in the apoptosis assay, a significant increase in the level of apoptosis was observed upon treatment with these NPs (Figure 4H), indicating that miR29A-B1, miR34A, and/or CASP8 can be effectively delivered to tumor spheroids via chitosan-PLGA NPs to induce apoptosis. Furthermore, the inhibition of BCL2 and SIRT1 target genes by miRs had a similar repression in tumor spheroids compared to A549 cells (Figures 4I, J).

The combination of miRs and CASP8 resulted in increased apoptosis, potentially due to enhanced CASP8 activity and restoration of lost apoptosis. Additionally, chitosan-PLGA NPs exhibited better penetration properties, as evidenced by the presence of Annexin-V-positive cells in the spheroid core. This study demonstrated that miR34A and miR29A-B1 can enhance CASP8 activity, leading to the arrest of tumor growth in both 2D and 3D models of NSCLC.

## 4 Conclusion

In conclusion, our study highlights the potential of chitosan-PLGA-based NPs for effective delivery of DNA cargo for cancer gene therapy. We have demonstrated that the combination of miR29A-B1 and miR34A with CASP8 can induce synergistic apoptosis in NSCLC cells, both in 2D and 3D models. Our findings open up promising possibilities for further optimization of chitosan-PLGA-NPs design for gene delivery and highlights their potential for translation into clinical settings.

## Data Availability

The original contributions presented in the study are included in the article/Supplementary Material, further inquiries can be directed to the corresponding author.
