# Supplementary material for: Apoptosis-targeted gene therapy for non-small cell lung cancer using chitosan-poly-lactic-co-glycolic acid -based nano-delivery system and CASP8 and miRs 29A-B1 and 34A
Source: Front Bioeng Biotechnol. 2023 Jun 6;11:1188652. doi: 10.3389/fbioe.2023.1188652 (PMC10281530; doi:10.3389/fbioe.2023.1188652)
Supplement: Supplementary file 1 [file DataSheet1.PDF]

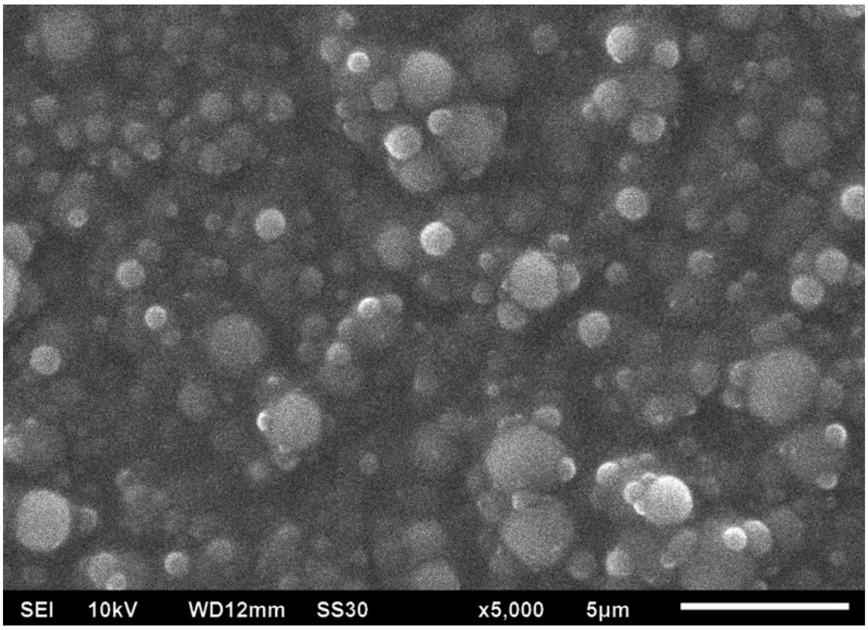

Supplementary Fig. S1: Scanning electron micrograph showing the size and distribution of the nanoparticles
